# 

**Published:** 1897-12

**Authors:** 


					﻿Vol. XVII.
DECEMBER, 1897.
NO. 12.
THE
HOMEOPATHIC pHYHlClAp,
A Monthly Journal of Homoeopathic Materia Medica
and Clinical Medicine.
“ He it a freeman whom truth makes free, and all are slaves beside.”
“Seek the Truth: come whence it may, cost what it will.”
EDITED AND PUBLISHED BY
WALTER M. JAMES, M. E>.
PHILADELPHIA :
TTo. 1231 LOCUST STREET.
X. O TT 3Z> O N ;
ALFRED IIEATII & CO., Sole Agf.nts for Great Britain,
114 Ebnry Street, Eaton Square.
Yearly Hubscri ytittn,	iiieui uibly in mlyiiiet, biiiyle nirmben, '•ill cis.
Entered at the Philadelphia Post-Office m Second-C)as< Mail Mauer.
				

